# Burning amazon: the dire consequences of climate inaction

**DOI:** 10.1016/j.lana.2024.100918

**Published:** 2024-10-11

**Authors:** The Lancet Regional Health – Americas

The devastating wildfires raging across South America demand our immediate attention. These fires are not merely natural disasters but stark manifestations of human-induced climate change and unsustainable exploitation of natural resources. The repercussions extend beyond immediate devastation, impacting human health, biodiversity, and the global climate. The catastrophic situation in the Amazon has turned what was once considered the world's lungs into the largest emitter of CO_2_, intensifying the global climate crisis and threatening the health of millions of people.

Between January and August, 2024, relentless fires have already devastated 11 million hectares in the Brazilian Amazon, an area almost as big as Guatemala. The peak of the fire season in the region is late September, however, a combination of factors resulted in an early start and a longer season in 2024. In July alone, 11,500 fires were recorded in the Brazilian Amazon, surging to 38,000 in August—the highest number in two decades. In Peru, the situation is equally dire, with forest fire alerts hitting a record high of over 1800 and at least 16 people dying due to the fires. Bolivia has recorded the highest number of fire points in the first half of any year: more than 17,700, 90% of which were recorded after July 1. In Ecuador, the mayor of Quito has described the city as being “under attack” from 27 fires, leading to the evacuation of more than 100 families. Presidents have declared a state of Emergency in Peru and Bolivia to allow for quick coordination of resources and international cooperation.

Small-scale farmers and Indigenous populations in South America have been using fire for land preparation for centuries, and it is a cultural and ancestral practice tolerated by many authorities. However, the uncontrolled deforestation accumulated in the past decades driven by mining and slash-and-burn agriculture for extensive agrobusiness has changed the landscape forever, making this practice a dangerous threat. Deforestation contributes to the changes in regional weather patterns that make dry seasons longer and more severe. Natural phenomena like El Niño, intensified by global warming, further weaken the Amazon rainforest, making it more vulnerable to destructive activities (such as mining, logging, and road constructions), amplifying droughts and fuelling this destructive feedback loop. Most countries suffering from wildfires are also experiencing their worst droughts in decades or even in their history. The historical drought in Brazil led to record low levels of the Amazon river and its affluents, impairing navigation, energy supply, and grain shipments across the region. In drier and hotter weather, forests become highly flammable, and fires—wherever they start—become uncontrollable. By contrast with previous years, many of this year's fires are not only burning farmlands and previously devastated areas but also primary forests, threatening the biodiversity of the Amazon and Pantanal wetlands and intensifying deforestation. Fire alerts in primary forests account for 61% of those in Peru and 70% in Bolivia and in many cases have been deemed intentional. Rodrigo Agostinho, president of the Brazilian Institute of Environment and Renewable Natural Resources, notes that invaders of public or demarcated lands are using fire instead of chainsaws, taking advantage of the drought to expand their lands illegally. Wildfires can be ignited by natural phenomena, such as lightning strikes, as we saw in Canada in 2023. However, very little lightning activity has been recorded in the region and most of the fires are believed to be human-provoked. Authorities from Peru and Brazil have explicitly linked the current situation to man-made fires, and dozens of people have been arrested in Quito, Argentina, and Brazil under suspicion of intentionally starting fires. On September 20, Brazilian President Luiz Inácio Lula da Silva signed a decree that raised fines for intentionally setting fires. Penalties can reach up to 10,000 Brazilian real (US$1826) per hectare burned. However, while punishing arsonists sends an important message, it does not even start to address the problem or contribute to the solution.

Enforcement of laws against illegal deforestation and arson will only be effective if paired with a complete shift from a reactive to a preventive approach against wildfires and a radical change in how we use the land. Preventive measures will need to incorporate public-facing campaigns to increase awareness of the dangers of forest fires to human, animal, and planetary health. Our traditional land use will need to change to prioritise sustainable use and management, eliminate slash-and-burn agriculture, halt deforestation, and protect primary forests and Indigenous territories, recognising the crucial role that these ecosystems have in global climate regulation. Investing in nature-based solutions can help mitigate the effects of climate change and build resilience. Reforestation projects, wetland restoration, and the protection of natural barriers can all serve to reduce the risk and severity of fires. According to a 2022 UN report, 50% of global investment goes into extinguishing wildfires and only 1% is directed at prevention and planning. As the world moves to a warmer and drier new normal, governments must not only redirect spending to prevention and planning but also recognise the importance of recovery and ecosystem resilience. Such strong commitment, however, has not yet been seen from any of the regional governments.

At the end of October, 2024, the UN Biodiversity Conference (COP 16) will take place in Cali, Colombia, and governments will review the state of implementation of the Kunming-Montreal Global Biodiversity Framework, designed to protect and restore the natural world, and its 23 action-oriented targets, including target 8, “Minimize the Impacts of Climate Change on Biodiversity and Build Resilience”. The high-level meeting is an opportunity for governments and stakeholders to acknowledge the failures in wildfire prevention and response strategies and commit to a radical shift in policies and practices.

The current emergency is a clear picture of how devastating the climate crisis can be if we refuse to change our exploitative model. South Americans are feeling the effects in their daily lives. The fires have dramatically reduced air quality across the region, pushing governments to mandate face mask use and stay-at-home warnings in many cities. São Paulo, the largest city in Latin America, was ranked the most polluted city in the world in early September because of fire smoke. We should leverage this tragic emergency to demand stricter climate change action plans to reduce greenhouse gas emissions, protect forests and biodiversity, and mitigate the impacts of global warming. The relentless destruction of the Amazon threatens not just neighbouring countries but the entire planet's ability to manage climate change. The cost of inaction is too high.

